# EXPLORING GENDER DISCORDANCE AND RISK OF CAREGIVING NEGLECT IN DEMENTIA CAREGIVING: A DYADIC PERSPECTIVE

**DOI:** 10.1093/geroni/igae098.2925

**Published:** 2024-12-31

**Authors:** David Hancock, E-Shien Chang, Sara Czaja

**Affiliations:** Weill Cornell Medicine, New York, New York, United States; Weill Cornell Medical College, New York, New York, United States; Weill Cornell Medicine/Center on Aging and Behavioral Research, New York, New York, United States

## Abstract

Neglect in caregiving relationships, defined as unmet Activities of Daily Living (ADL) or Instrumental Activities of Daily Living (IADL) needs, remains a critical impediment undermining the safety of persons living with dementia (PLWD). This study explores the occurrence of neglect in gender-concordant and discordant caregiving dyads, where the caregiver and care recipient are of same or different genders. Data were drawn from 244 dyads of racially/ethnically diverse caregivers (CR) and PLWD. We operationalized neglect as CG’s failure to meet any of the 6 ADL or 8 IADL for PLWD. Chi-squared tests were employed to compare neglect occurrences across three groups: female-male, male- female, and same-gender dyads. Overall, female-male caregiving dyads were most common in our study sample (n=103), followed by same gender dyads (n=102), and male-female dyads (n=39). The chi-square test indicated significant differences in neglect occurrences across the three groups (p<.05). Specifically, male caregivers caring for female care recipients exhibited more instances of one-week prevalence of neglect. ADL/IADL item-level analyses did not reach statistical significance due to small sample sizes; they generally supported the overall pattern. The findings suggest that gender concordance and discordance between CG and PLWD may be an important area for dementia caregiving intervention research. Future studies should focus on vulnerabilities and strengths of male CG caring for female PLWD to better support and mitigate risks of neglect in this population. These results have implications for policy and caregiving research, emphasizing the need to consider gender dynamics in caregiver support and training programs.

